# In Vitro Evaluation of Disinfectants on Gutta-Percha Cones: Antimicrobial Efficacy Against *Enterococcus faecalis* and *Candida albicans*

**DOI:** 10.3390/jcm14196846

**Published:** 2025-09-27

**Authors:** Tringa Kelmendi, Donika Bajrami Shabani, Aida Meto, Hani Ounsi

**Affiliations:** 1 Department of Dental Pathology and Endodontics, Faculty of Medicine, University of Prishtina, 10000 Prishtina, Kosovo; tringa.kelmendi@uni-pr.edu; 2 Department of Dentistry, Faculty of Dental Sciences, University of Aldent, 1007 Tirana, Albania; 3 Laboratory of Microbiology and Virology, Department of Surgery, Medicine, Dentistry and Morphological Sciences with Interest in Transplant, Oncology, and Regenerative Medicine, University of Modena and Reggio, Emilia, 41125 Modena, Italy; 4 Department of Conservative Dentistry and Endodontics, Dr. D.Y. Patil Dental College and Hospital, Dr. D.Y. Patil Vidyapeeth, Pimpri, Pune 411018, Maharashtra, India; 5 Department of Medical Biotechnologies, University of Siena, 53100 Siena, Italy; ounsih@gmail.com

**Keywords:** *Candida albicans*, chlorhexidine, disinfection, endodontics, *Enterococcus faecalis*, gutta-percha cones, sodium hypochlorite

## Abstract

**Background/Objectives:** Periradicular disease is largely microbial in origin. Even gutta-percha (GP) cones manufactured under aseptic conditions can acquire contaminants during handling or storage, undermining otherwise adequate canal preparation. To assess residual antimicrobial activity on GP cones after brief exposure to five endodontic disinfectants: sodium hypochlorite (NaOCl) 1%, 2.5%, 5.25%; chlorhexidine (CHX) 2%; and glutaraldehyde 2% against *Enterococcus faecalis* and *Candida albicans*. **Methods:** Standardized GP cones were dipped for 5–120 s, blotted on neutralizing gauze, and placed on agar inoculated with either organism. Using an agar diffusion approach, inhibition-zone diameters were recorded at 0, 24, and 48 h. Data were summarized using descriptive statistics (means, standard deviations, and 95% confidence intervals) for each disinfectant–dip-time combination. **Results:** By 24 h, inhibition zones were observed for most disinfectants; for *C. albicans,* glutaraldehyde 2% showed no measurable effect. At later time points, performance depended on both disinfectant and contact time. For *E. faecalis*, NaOCl 2.5% and 5.25% yielded the largest zones at 48 h (20–21 mm at 120 s), whereas NaOCl 1% was smaller (10 mm) and glutaraldehyde 2% modest (9 mm). For *C. albicans*, NaOCl 2.5% and CHX 2% were most effective at 48 h (17–19 mm at 120 s); NaOCl 5.25% was intermediate, NaOCl 1% weak, and glutaraldehyde 2% showed no measurable antifungal effect. Longer immersions (≥45 s) consistently increased inhibition zone diameters. **Conclusions:** Residual antimicrobial activity on GP cones depends on both the agent and the immersion time. For *E. faecalis,* higher concentration NaOCl produced the largest zones at short contact time, whereas for *C. albicans,* CHX 2% and NaOCl 2.5% provided the most reliable carryover. Selecting an appropriate concentration and allowing sufficient dip time may reduce reinfection risk at obturation.

## 1. Introduction

The primary objective of endodontic treatment is to eliminate microbial pathogens and prevent reinfection of the canal [1]. For effective infection control in endodontics, every instrument and material that enters the root canal should be free of microorganisms. In daily practice, gutta-percha (GP) remains the main choice for obturation because it is easy to handle, dimensionally stable, and generally well-tolerated by tissues [2,3]. Even though the cones come in sealed packages, they are not completely sterile, and once the package is opened, they can easily be contaminated. Some studies have detected microbial growth even in cones taken from newly opened boxes [2]. This highlights why a disinfection step is necessary before these cones are used on patients. Conventional sterilization methods, such as moist or dry heat, are not suitable for GP due to concerns about structural integrity [3]. In a busy clinical setting, even brief exposure to gloves, instruments, or surrounding surfaces may introduce microorganisms that compromise the cleanliness of the root canal right before obturation. This poses a risk of introducing bacteria or fungi into the canal during obturation, which could compromise treatment outcomes [4].

Persistent endodontic infections are frequently associated with resilient microorganisms such as *Enterococcus faecalis* and *Candida albicans* [5,6]. Both *E. faecalis* and *C. albicans* have been linked to recurrent infections and endodontic failure, largely due to their resistance to disinfectants, ability to form biofilms, and capacity to invade dentin [7,8], characteristics that can contribute to less favorable clinical outcomes [9,10]. To reduce the risk of contaminating the root canal system during obturation, various chemical solutions have been tested for disinfecting GP cones chairside. Among them, sodium hypochlorite (NaOCl) remains one of the most commonly used agents. Its antimicrobial effectiveness is well established, particularly at higher concentrations (2.5% to 5.25%), and it also has the added advantage of dissolving organic tissue [11]. Nevertheless, its potential to alter the physical integrity of GP raises concerns [12]. Chlorhexidine gluconate (CHX), by contrast, is valued for its substantivity and relatively lower impact on GP properties [13]. Glutaraldehyde is not commonly used in endodontics, but its effectiveness as a high-level disinfectant has been well established in broader medical practice, and in recent years it has also attracted attention for possible dental applications [14].

Although many studies have evaluated individual disinfectants, only a limited number have compared their performance against both *E. faecalis* and *C. albicans* at different exposure times. There is still no clear agreement on how long GP cones should remain in disinfectant solutions to achieve effective microbial control without compromising their physical properties [15,16].

The main aim of this study was to evaluate and compare the short-term antimicrobial effectiveness of five commonly used disinfectants, NaOCl (at concentrations of 1%, 2.5%, and 5.25%), CHX 2%, and glutaraldehyde 2%, against *E. faecalis* and *C. albicans* in an agar diffusion model. The null hypothesis was that all tested disinfectants would exhibit equal antimicrobial effectiveness against *E. faecalis* and *C. albicans*, regardless of concentration or exposure time.

## 2. Materials and Methods

### 2.1. GP Sample Preparation

A total of 1050 standardized GP cones (#25 and #30, ISO color coding; VDW GmbH, Munich, Germany), from a single manufacturer, were used to ensure uniform product characteristics and packaging. Cones were removed directly from sealed manufacturer packs using sterile tweezers, previously autoclaved, under aseptic laboratory conditions to prevent contamination. The cones were randomly divided into seven groups (*n* = 150) according to the disinfectant to be tested.

### 2.2. Disinfectants

Five disinfectant solutions used in endodontics were evaluated: NaOCl at 1%, 2.5%, and 5.25%; CHX 2%; and glutaraldehyde 2%. Two controls were included as described in Section 2.5. All solutions were used at room temperature (approximately 22–24 °C) as supplied by the manufacturers; pH was not measured. The disinfectants, their concentrations, and manufacturers are summarized in Table 1.

### 2.3. Experimental Design and Exposure Protocol

**Chart 1:**
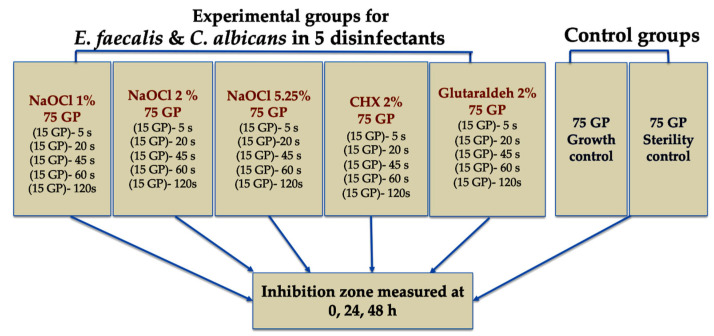
Allocation of the GP cones into disinfectant/control groups, microorganism subgroups, and immersion time sets.

Each disinfectant group (*n* = 150) was divided into two subgroups (*n* = 75 each) for *E. faecalis* (ATCC 29212, Liofilchem, Italy) or *C. albicans* (ATCC 10231, Liofilchem, Italy). Each subgroup was divided into five exposure times (*n* = 15: 5, 20, 45, 60, and 120 s. Sterile cones were immersed in the assigned disinfectant for the specified time, removed aseptically, lightly blotted, and placed on inoculated agar plates. For *E. faecalis*, blood agar (nutrient agar with 5% defibrinated sheep blood) was used; for *C. albicans*, Mueller–Hinton agar. Inoculum density was adjusted to approximately 0.5 McFarland, and a uniform lawn was prepared by swabbing in three directions. Plates were allowed to dry for 10–15 min before cone placement. Cones were positioned radially, spaced ≥ 20 mm apart and ≥15 mm from the plate edge. Plates were incubated aerobically at 37 °C and read at 0, 24, and 48 h for growth inhibition zones, according to the agar diffusion assay (Chart 1).

### 2.4. Evaluation of Antimicrobial Activity (Agar Diffusion-Based Assay)

The standard agar diffusion test [17] was performed with minor modifications to assess residual antimicrobial activity retained by the disinfectant-treated cones. Briefly, cones exposed to the various disinfectants for different times were placed onto freshly inoculated agar plates (either *E. faecalis* or *C. albicans*). Before transfer, cones were briefly blotted on sterile gauze moistened with a neutralizing solution (for example, 0.5% sodium thiosulfate for NaOCl and sterile saline for CHX and glutaraldehyde) to minimize carry-over artefacts from residual disinfectant. Growth inhibition zones (clear areas around each cone) were measured in millimetres at 0, 24, and 48 h using a calibrated digital calliper. For non-circular halos, the mean of two perpendicular diameters was recorded. Measurements were performed under blinded conditions to reduce bias. Placing disinfectant-treated cones onto inoculated agar was chosen solely to evaluate residual antimicrobial activity retained on the cone surface after brief chemical exposure, not to measure pre-existing contamination.

### 2.5. Control Validation

A sterility control consisted of sterile GP cones placed on uninoculated agar to confirm the absence of contamination. A growth control consisted of cones exposed only to sterile saline before placement on inoculated agar.

### 2.6. Statistical Analysis

Fifteen GP cones were measured for each disinfectant–dip-time combination (*n* = 15 per group). Zone diameters are summarized descriptively as mean, standard deviation, minimum, maximum, and 95% confidence intervals for each combination. No formal inferential hypothesis test or between-group significance testing was performed. Calculations were performed in SPSS Statistics, version 21 (IBM Corp., Armonk, NY, USA). Line plots were created to illustrate trends across dip times and disinfectants.

### 2.7. Ethical Consideration

This in vitro study used standard laboratory materials only; therefore, ethical approval was not applicable.

## 3. Results

### 3.1. Antimicrobial Activity Against E. faecalis

The diffusion assay was performed as described. Sterility and growth controls behaved as expected, confirming cone sterility and the absence of intrinsic antimicrobial activity of GP. Where zones were present at 24 h, they remained visible at 48 h, as detailed in Table 2. Zone diameters varied by disinfectant and immersion time; very short dips (5–20 s) produced little or no effect, except for small halos for 5.25% NaOCl. Once exposure reached 45–60 s, clear zones appeared, and the largest diameters were recorded at 120 s. At 48 h after a 120 s dip (*n* = 15), the mean ± SD (min–max) were 20.9 ± 0.4 mm (20–21) for 5.25% NaOCl, 19.9 ± 0.4 mm (19–20) for 2.5% NaOCl, 12.7 ± 0.5 mm (12–13) for 2% CHX, 9.1 ± 0.3 mm (9–10) for 2% glutaraldehyde, and 9.6 ± 0.6 mm (8–10) for 1% NaOCl; values at 48 h were slightly lower than at 24 h, but the rank order was unchanged.

### 3.2. Antimicrobial Activity Against C. albicans

In the agar-diffusion assay with *C. albicans* (Table 3), short immersions of 5–20 s produced no measurable zones for any agent. Once exposure reached 45–60 s, inhibition became evident and peaked at 120 s. At 48 h after a 120 s dip (*n* = 15), the mean diameters ± SD (min–max) were 18.8 ± 0.4 mm (18–19) for 2.5% NaOCl and 16.7 ± 0.5 mm (16–17) for 2% CHX; 5.25% NaOCl showed an intermediate effect at 13.0 ± 0.0 mm (13–13), 1% NaOCl was weak at 5.7 ± 0.5 mm (5–6), and 2% glutaraldehyde showed no zones at any time point. This pattern indicates that, beyond the 45–60 s threshold, extended contact yields clear antifungal carryover, with 2.5% NaOCl and 2% CHX providing the strongest effects, 5.25% NaOCl a moderate effect, and 1% NaOCl a limited one, while glutaraldehyde remains inactive.

### 3.3. Relevance of the Dip Times

**Figure 1:**
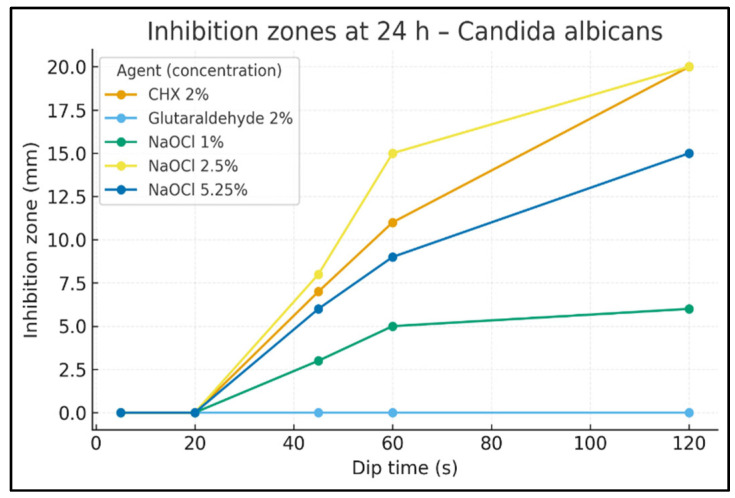
Inhibition zones at 24 h—*C. albicans*. Line plots show inhibition-zone diameter (mm) versus dip time (s) for CHX 2%, Glutaraldehyde 2%, NaOCl 1%, NaOCl 2.5%, and NaOCl 5.25%. Data are descriptive (no hypothesis testing).

**Figure 2:**
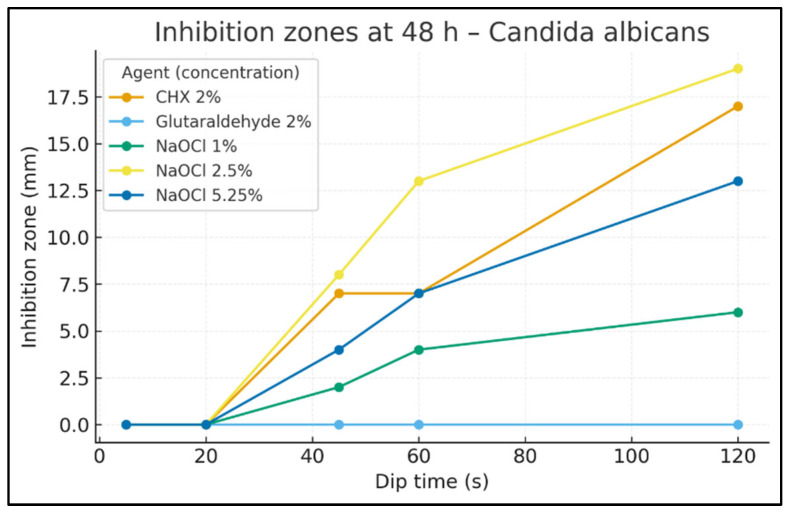
Inhibition zones at 48 h—*C. albicans*. Inhibition-zone diameter (mm) by dip time (s) for CHX 2%, Glutaraldehyde 2%, NaOCl 1%, NaOCl 2.5%, and NaOCl 5.25%. Data are descriptive (no hypothesis testing).

**Figure 3:**
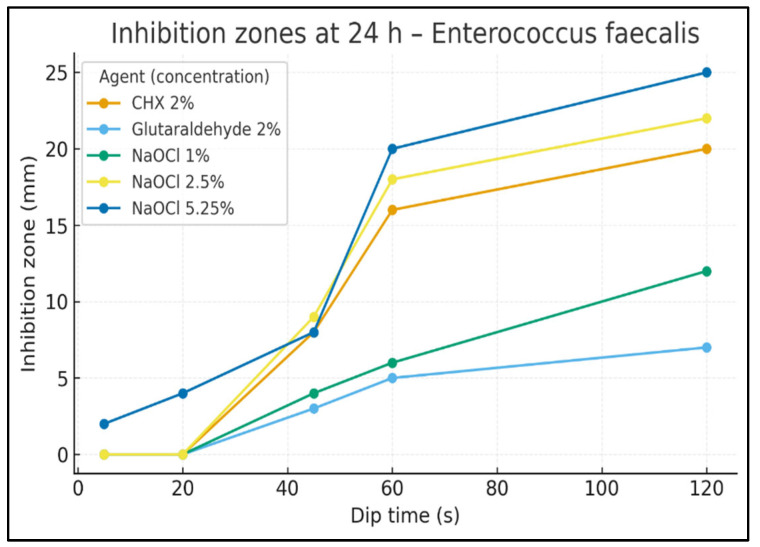
Inhibition zones at 24 h—*E. faecalis*. Inhibition-zone diameter (mm) vs. dip time (s) for CHX 2%, Glutaraldehyde 2%, NaOCl 1%, NaOCl 2.5%, and NaOCl 5.25%. Data are descriptive (no hypothesis testing).

**Figure 4:**
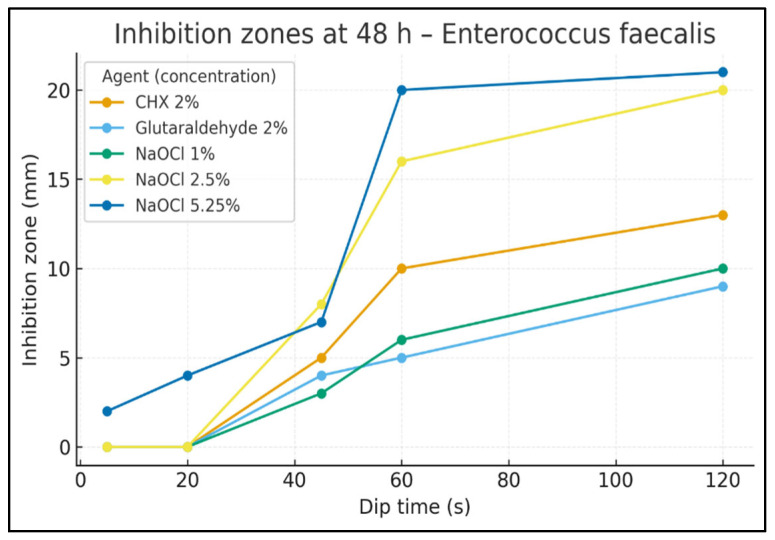
Inhibition zones at 48 h—*E. faecalis*. Inhibition-zone diameter (mm) vs. dip time (s) for CHX 2%, Glutaraldehyde 2%, NaOCl 1%, NaOCl 2.5%, and NaOCl 5.25%. Data are descriptive (no hypothesis testing).

Figure 1, Figure 2, Figure 3 and Figure 4 show how cone exposure time affected residual antifungal and antibacterial activity.

At 24 h (Figure 1), dip times ≤ 20 s produced no halos for any agent. At 45 s, activity was evident for NaOCl 2.5% (8 mm) and CHX 2% (7 mm), with smaller zones for NaOCl 5.25% (6 mm) and NaOCl 1% (3 mm); glutaraldehyde 2% stayed inactive (0 mm). At 60 s, the pattern intensified: NaOCl 2.5% reached 15 mm, CHX 11 mm, NaOCl 5.25% 9 mm, NaOCl 1% 5 mm, and glutaraldehyde 0 mm. By 120 s, the largest halos were 20 mm for both NaOCl 2.5% and CHX 2%, followed by NaOCl 5.25% (15 mm) and NaOCl 1% (6 mm); glutaraldehyde remained 0 mm.

At 48 h (Figure 2), the time–response persisted but with slightly different magnitudes. Halos were absent at 20 s. At 45 s, zones measured 8 mm for NaOCl 2.5%, 7 mm for CHX 2%, 4 mm for NaOCl 5.25%, 2 mm for NaOCl 1%, and 0 mm for glutaraldehyde. At 60 s, NaOCl 2.5% was 13 mm, NaOCl 5.25% 7 mm, CHX 2% stayed 7 mm, NaOCl 1% 4 mm, and glutaraldehyde 0 mm. By 120 s, NaOCl 2.5% was highest at 19 mm, CHX 2% 17 mm, NaOCl 5.25% 13 mm, NaOCl 1% 6 mm, and glutaraldehyde 0 mm.

At 24 h (Figure 3), a small early effect was already visible for NaOCl 5.25% at 20 s (4 mm), while other agents were inactive. At 45 s, halos measured 9 mm for NaOCl 2.5%, 8 mm for CHX 2% and NaOCl 5.25%, 4 mm for NaOCl 1%, and 3 mm for glutaraldehyde. At 60 s, NaOCl 5.25% rose to 20 mm, NaOCl 2.5% 18 mm, CHX 16 mm, NaOCl 1% 6 mm, and glutaraldehyde 5 mm. At 120 s, NaOCl 5.25% was highest at 25 mm, followed by NaOCl 2.5% (22 mm), CHX (20 mm), NaOCl 1% (12 mm), and glutaraldehyde (7 mm).

At 48 h (Figure 4), NaOCl 5.25% again showed measurable inhibition as early as 20 s (4 mm), whereas other agents remained at 0 mm. At 45 s, zones were 8 mm for NaOCl 2.5%, 7 mm for NaOCl 5.25%, 5 mm for CHX 2%, 4 mm for glutaraldehyde, and 3 mm for NaOCl 1%. At 60 s, NaOCl 5.25% reached 20 mm, NaOCl 2.5% 16 mm, CHX 10 mm, NaOCl 1% 6 mm, and glutaraldehyde 5 mm. At 120 s, the final diameters were 21 mm for NaOCl 5.25%, 20 mm for NaOCl 2.5%, 13 mm for CHX 2%, 10 mm for NaOCl 1%, and 9 mm for glutaraldehyde.

Dip times below 20 s were ineffective except for a small early effect of NaOCl 5.25% against *E. faecalis*. The steepest increases occurred between 45 s and 60 s, with near-maximal halos by 120 s. NaOCl 2.5% and CHX 2% were the most reliable against *C. albicans*, while NaOCl 5.25% consistently produced the largest zones against *E. faecalis*.

## 4. Discussion

This study evaluated the short-term residual antimicrobial activity of five endodontic disinfectants that had been used to treat GP cones at different concentrations and for different times. The disinfectant-treated GP cones were then analyzed by an agar diffusion-based assay. Differences in residual antimicrobial effects emerged at 24 and 48 h across agents and immersion times. Patterns were consistent with the descriptive data: for *E. faecalis*, NaOCl 2.5% and 5.25% produced the largest inhibition zones at 48 h, CHX 2% was intermediate, whereas NaOCl 1% and glutaraldehyde 2% were little or not effective. For *C. albicans*, CHX 2% and NaOCl 2.5% were the most effective, NaOCl 5.25% was intermediate, NaOCl 1% was weak, and glutaraldehyde 2% showed no measurable effect.

NaOCl’s strong early antimicrobial action but uncertain lasting effect has been noted by other authors [17,18,19,20], and our findings fit that pattern. In our plates, the halos from higher-strength NaOCl tended to shrink by 24–48 h, which could be explained by biofilm-related tolerance or adaptive responses described by Arias-Moliz et al. (2015) and Fiegler-Rudol et al. (2025) [15,21]. Interestingly, NaOCl at 1% behaved more steadily over time, a result that may indicate a better balance between microbial suppression and preservation of gutta-percha integrity, in line with Mishra et al. (2024) [22]. Although NaOCl is highly effective, its caustic nature and the risk of tissue damage if extruded beyond the apex, particularly at higher concentrations, mean that careful handling is essential in clinical practice [23].

CHX at 2% performed reliably against *E. faecalis* but showed inconsistent antifungal effects against *C. albicans*. The variability in inhibition-zone diameters at 24 and 48 h may reflect CHX’s limited penetration into fungal biofilms and its reduced activity in the presence of organic material, as reported by Dudás et al. (2025), Antonelli et al. (2019), and Rossi-Fedele et al. (2011) [24,25,26]. Despite these limitations, CHX produced intermediate but comparatively stable antibacterial halos, reinforcing its usefulness as a steady antibacterial agent rather than a primary antifungal disinfectant [12,13]. In contrast, glutaraldehyde 2% produced only modest inhibition against *E. faecalis* and no measurable effect against *C. albicans* in this agar-diffusion model. This outcome contrasts with its well-established role as a high-level disinfectant in broader medical practice [14,15] and with prior suggestions of its potential chairside use in endodontics [27,28]; further work in dentin or biofilm models is needed to clarify any residual benefits. Across all groups, *E. faecalis* was more susceptible than *C. albicans*, underscoring the latter’s clinical relevance in persistent endodontic infections [5,6]. Disinfectant selection and immersion time should therefore be tailored to the clinical scenario. In our agar-diffusion model, NaOCl 2.5–5.25% produced the largest residual inhibition zones for *E. faecalis* at 48 h, whereas for C. albicans, CHX 2% and NaOCl 2.5% performed best; NaOCl 5.25% was intermediate, NaOCl 1% weak, and glutaraldehyde 2% showed no measurable antifungal effect. CHX can serve as a useful adjunct for bacterial contamination but may require extended exposure or combination strategies for antifungal coverage [24,25,26]. Claims of prolonged suppression with glutaraldehyde 2% or 1% NaOCl are not supported by our data and, if mentioned based on external reports, should be presented as literature context rather than conclusions from this study [22,27,28].

This study has limitations that must be acknowledged. The in vitro design cannot fully replicate the complexity of clinical conditions, including anatomical variations, the buffering effects of dentin, and host immune responses. The agar diffusion method, while standardized and reproducible, does not adequately simulate biofilm resistance or agent penetration into dentin. Incubation was controlled at 37 °C. Furthermore, during the in vitro disinfection step, solution temperature and pH were not controlled, and no mechanical agitation was applied, although all these factors can enhance disinfectant penetration in clinical practice [29]. Possible carryover effects from residual disinfectant cannot be entirely excluded, even after blotting/neutralization. The biochemical properties of each disinfectant and the choice of the culture media may also have influenced halo sizes. Beyond antimicrobial carryover, in vitro studies have shown that disinfecting solutions can alter GP surface properties; because we did not assess surface integrity (e.g., via SEM/FTIR), our clinical recommendations should be interpreted with this limitation in mind [8,22,26].

In future work, researchers could employ molecular tools such as quantitative PCR (qPCR) or confocal microscopy to examine microbial survival and agent penetration into dentin more directly. Other methods, including scanning electron microscopy or infrared spectroscopy, could reveal how these chemicals affect the surface or composition of GP cones [3]. It would also be worthwhile to explore combination approaches, using NaOCl followed by CHX or newer tools like nanoparticle-based disinfectants and photodynamic therapy. Such studies could help strike a better balance between eliminating microbes and preserving cone integrity, making chairside disinfection both safer and more dependable.

## 5. Conclusions

Within the limits of this in vitro model, most disinfectants produced measurable halo zones by 24 h, with size depending on the agent and the contact time. For *E. faecalis*, NaOCl 2.5% and 5.25% led at 48 h; for *C. albicans*, CHX 2% and NaOCl 2.5% performed best, while glutaraldehyde 2% showed no antifungal effect. These findings open up dentin/biofilm models as a further step towards clinical practice changes.

## Figures and Tables

**Table 1:** Disinfectants evaluated in the study, including concentrations and manufacturers.

Groups	Disinfectants	Concentrations	Manufacturers
1	CHX	2%	Cerkamed, Stalowa Wola, Poland
2	NaOCl	1%	Cerkamed, Stalowa Wola, Poland
3	NaOCl	2.5%	Merck, Darmstadt, Germany
4	NaOCl	5.25%	Cerkamed, Stalowa Wola, Poland
5	Glutaraldehyde	2%	Glutarex, 3M Company, Bangalore, India
6	Sterility control	-	Sterile saline; cones placed on uninoculated agar (Factormed, Agualva-Cacém, Portugal)
7	Growth control	-	Sterile saline; cones placed on inoculated agar (Factormed, Agualva-Cacém, Portugal)

**Table 2:** Inhibition-zone diameters (mm) at 24 h and 48 h for *E. faecalis* by disinfectant and immersion time. GP: gutta-percha; SD: standard deviation.

*E. faecalis*			
Disinfectant	Dip Time (s) *n* (GP Cones) = 15 per Group	24 h (mm) Mean ± SD (Min–Max)	48 h (mm) Mean ± SD (Min–Max)
1% NaOCl	5	0.0 (0.0–0.0)	0.0 (0.0–0.0)
1% NaOCl	20	0.0 (0.0–0.0)	0.0 (0.0–0.0)
1% NaOCl	45	3.9 ± 0.7 (3.0–5.0)	2.9 ± 0.7 (2.0–4.0)
1% NaOCl	60	5.5 ± 0.7 (4.0–6.0)	5.5 ± 0.7 (4.0–6.0)
1% NaOCl	120	11.6 ± 0.6 (10.0–12.0)	9.6 ± 0.6 (8.0–10.0)
2.5% NaOCl	5	0.0 (0.0–0.0)	0.0 (0.0–0.0)
2.5% NaOCl	20	0.0 (0.0–0.0)	0.0 (0.0–0.0)
2.5% NaOCl	45	8.7 ± 0.5 (8.0–9.0)	7.7 ± 0.5 (7.0–8.0)
2.5% NaOCl	60	17.7 ± 0.6 (16.0–18.0)	15.7 ± 0.6 (14.0–16.0)
2.5% NaOCl	120	21.9 ± 0.4 (21.0–22.0)	19.9 ± 0.4 (19.0–20.0)
5.25% NaOCl	5	1.8 ± 0.4 (1.0–2.0)	1.8 ± 0.4 (1.0–2.0)
5.25% NaOCl	20	3.9 ± 0.4 (3.0–4.0)	3.9 ± 0.4 (3.0–4.0)
5.25% NaOCl	45	8.1 ± 0.6 (7.0–9.0)	7.1 ± 0.6 (6.0–8.0)
5.25% NaOCl	60	19.6 ± 0.7 (18.0–20.0)	19.6 ± 0.7 (18.0–20.0)
5.25% NaOCl	120	24.9 ± 0.4 (24.0–25.0)	20.9 ± 0.4 (20.0–21.0)
2% CHX	5	0.0 (0.0–0.0)	0.0 (0.0–0.0)
2% CHX	20	0.0 (0.0–0.0)	0.0 (0.0–0.0)
2% CHX	45	8.1 ± 0.6 (7.0–9.0)	5.0 ± 0.5 (4.0–6.0)
2% CHX	60	15.5 ± 0.8 (14.0–16.0)	9.6 ± 0.5 (9.0–10.0)
2% CHX	120	19.2 ± 1.2 (17.0–20.0)	12.7 ± 0.5 (12.0–13.0)
2% Glutaraldehyde	5	0.0 (0.0–0.0)	0.0 (0.0–0.0)
2% Glutaraldehyde	20	0.0 (0.0–0.0)	0.0 (0.0–0.0)
2% Glutaraldehyde	45	3.5 ± 0.5 (3.0–4.0)	3.7 ± 0.5 (3.0–4.0)
2% Glutaraldehyde	60	5.4 ± 0.5 (5.0–6.0)	5.4 ± 0.5 (5.0–6.0)
2% Glutaraldehyde	120	7.2 ± 0.4 (7.0–8.0)	9.1 ± 0.3 (9.0–10.0)

The colors are used to distinguish between the groups tested with each disinfectant.

**Table 3:** Inhibition-zone diameters (mm) at 24 h and 48 h for *C. albicans* by disinfectant and immersion time. GP: gutta-percha; SD: standard deviation.

*C. albicans*			
Disinfectant	Dip Time (s) n (GP Cones) = 15 per Group	24 h (mm) Mean ± SD (Min–Max)	48 h (mm) Mean ± SD (Min–Max)
1% NaOCl	5	0.0 (0.0–0.0)	0.0 (0.0–0.0)
1% NaOCl	20	0.0 (0.0–0.0)	0.0 (0.0–0.0)
1% NaOCl	45	2.3 ± 0.8 (1.0–3.0)	1.3 ± 0.8 (0.0–2.0)
1% NaOCl	60	4.3 ± 1.0 (2.0–5.0)	3.3 ± 1.0 (1.0–4.0)
1% NaOCl	120	6.0 ± 0.0 (6.0–6.0)	5.7 ± 0.5 (5.0–6.0)
2.5% NaOCl	5	0.0 (0.0–0.0)	0.0 (0.0–0.0)
2.5% NaOCl	20	0.0 (0.0–0.0)	0.0 (0.0–0.0)
2.5% NaOCl	45	8.0 ± 0.0 (8.0–8.0)	8.0 ± 0.0 (8.0–8.0)
2.5% NaOCl	60	14.7 ± 0.5 (14.0–15.0)	12.7 ± 0.5 (12.0–13.0)
2.5% NaOCl	120	19.8 ± 0.4 (19.0–20.0)	18.8 ± 0.4 (18.0–19.0)
5.25% NaOCl	5	0.0 (0.0–0.0)	0.0 (0.0–0.0)
5.25% NaOCl	20	0.0 (0.0–0.0)	0.0 (0.0–0.0)
5.25% NaOCl	45	6.1 ± 0.3 (6.0–7.0)	4.1 ± 0.3 (4.0–5.0)
5.25% NaOCl	60	8.5 ± 0.8 (7.0–9.0)	6.7 ± 0.5 (6.0–7.0)
5.25% NaOCl	120	15.0 ± 0.0 (15.0–15.0)	13.0 ± 0.0 (13.0–13.0)
2% CHX	5	0.0 (0.0–0.0)	0.0 (0.0–0.0)
2% CHX	20	0.0 (0.0–0.0)	0.0 (0.0–0.0)
2% CHX	45	7.2 ± 0.4 (7.0–8.0)	7.0 ± 0.0 (7.0–7.0)
2% CHX	60	10.5 ± 0.8 (9.0–11.0)	6.7 ± 0.5 (6.0–7.0)
2% CHX	120	19.7 ± 0.5 (19.0–20.0)	16.7 ± 0.5 (16.0–17.0)
2% Glutaraldehyde	5	0.0 (0.0–0.0)	0.0 (0.0–0.0)
2% Glutaraldehyde	20	0.0 (0.0–0.0)	0.0 (0.0–0.0)
2% Glutaraldehyde	45	0.0 (0.0–0.0)	0.0 (0.0–0.0)
2% Glutaraldehyde	60	0.0 (0.0–0.0)	0.0 (0.0–0.0)
2% Glutaraldehyde	120	0.0 (0.0–0.0)	0.0 (0.0–0.0)

The colors are used to distinguish between the groups tested with each disinfectant.

## Data Availability

The data presented in this study are available on request from the corresponding authors.
